# Evaluation of a Novel Precision Biotic on Enterohepatic Health Markers and Growth Performance of Broiler Chickens under Enteric Challenge

**DOI:** 10.3390/ani12192502

**Published:** 2022-09-20

**Authors:** Britt Blokker, Cristiano Bortoluzzi, Christelle Iaconis, Estefania Perez-Calvo, Maria C. Walsh, Ghislain Schyns, Ian Tamburini, Jack M. Geremia

**Affiliations:** 1 DSM Nutritional Products, 4303 Kaiseraugst, Switzerland; 2 DSM Nutritional Products, 68128 Village-Neuf, France

**Keywords:** broiler, intestinal health, microbiome metabolic modulator, inflammation

## Abstract

**Simple Summary:**

Precision biotics (PB) are nutritional products that influence targeted metabolic pathways of the microbiome to control the profile of metabolites produced in the gut by the bacteria, such as short chain fatty acids (SCFA) and nitrogen-related metabolites. The objective of the studies presented herein was to evaluate the effect of feeding PB to broiler chickens on the response against enteric stress. It was observed that the PB improved the intestinal health of experimentally challenged chickens, and the growth performance of chickens undergoing a natural enteric challenge under commercial-like conditions. The beneficial action of this PB on the microbiome pathways may explain the enhanced markers of intestinal health, such as intestinal histology, expression of nutrient transporter, inflammation, and cell cycling-related genes.

**Abstract:**

This study evaluated the supplementation of a precision biotic (PB) on the enterohepatic health markers and growth performance of broiler chickens undergoing an enteric challenge. In the first study, three treatments were used: Unchallenged Control (UC); Challenged Control (CC; dietary challenge and 10× dose of coccidia vaccine); and a challenged group supplemented with PB (1.3 kg/ton). In the second study, three treatments were used: control diet, diet supplemented with Avilamycin (10 ppm), and a diet supplemented with PB (0.9 kg/ton). All the birds were exposed to natural challenge composed by dietary formulation and reused litter from a coccidiosis positive flock. In Trial 1, PB decreased ileal histological damage, increased villi length, and the expression of *SLC5A8* in ileal tissue versus CC; it reduced ileal expression of *IL-1β* compared to both UC and CC treatments. PB increased the expression of cell cycling gene markers *CCNA2* and *CDK2* in the ileum compared to CC. In Trial 2, PB improved the growth performance, intestinal lesion scores and intestinal morphology of broiler chickens. These results indicate that birds supplemented with PB are more resilient to enteric challenges, probably by its action in modulating microbiome metabolic pathways related to nitrogen metabolism and protein utilization.

## 1. Introduction

In the last decades, significant advances in molecular biology, analytics, and data science have dramatically increased our understanding of the functional pathways of the gastrointestinal microbiome of humans and animals [1,2,3]. These advances have led to the development of precision biotics (PB), a new class of microbiome metabolic modulators that influence targeted metagenomic functions of the microbiome [4]. Precision biotics control the output of metabolites by the gut microbiome to deliver beneficial outcomes to the animal and the environment [5].

The concept of targeting microbiome pathways to achieve performance and health outcomes relies on two fundamental principles. The first is the existence of core microbiome pathways for energy, amino acid, and nucleotide metabolism across microorganisms [6]. The second is the recognition that these core microbiome pathways are highly conserved, even across diverse populations of individuals. The latter property lies in stark contrast to taxonomic composition (e.g., the gut microbial profile), which often varies significantly across a population of otherwise similar individuals [7]. These insights suggest that targeting core microbiome pathways, rather than working to adjust the microbial profile, offers the potential for improved consistency [4].

One strategy for developing PB is by screening tailored glycans for their ability to modulate selected microbiome pathways across a diverse set of microbial communities [8,9]. Glycans provide a chemically diverse class of molecular structures known to exhibit a wide array of bioactivities on microbial communities [10,11]. However, the detailed way glycans affect the microbiome metabolic function is linked tightly to its structure. For example, many prebiotics, such as those obtained from plant fiber, pectins, and other agricultural sources (including those obtained by partial enzymatic hydrolysis), are not developed to target selected metagenomic functions. In contrast to many prebiotics and probiotics used in animal nutrition, the potential of PB is their ability to activate core microbial pathways to achieve consistency, rather than by modulating the abundance of microbial taxa that vary from animal to animal [12].

The ability to steer microbiome pathways in the gastrointestinal tract (GIT) of animals with the use of PB opens immense possibilities to modulate host physiology through microbial metabolites. The immune system is a notable example of the symbiotic relationship between the host and its microbiome, where the normal immune development and function of the host depends on a diverse array of microbial metabolites produced from dietary and host substrates and synthesized de novo by the microbiota [13]. This close symbiotic relationship has given way to the idea of the holo-biont, as an organism composed of the host and its microbiome [14]. However, when this symbiotic relationship is disturbed by enteric infections, such as coccidiosis in broiler chickens, the use of PB to redirect microbial metabolic functions may be an important nutritional strategy to minimize the negative impact of such infections.

Therefore, the objective of the present studies was to evaluate the effects of a PB, selected for its ability to increase the metabolic output of propionate and butyrate biosynthesis pathways and modulate amino acid degradation and amine metabolism, on enterohepatic health markers of broiler chickens using an intestinal inflammation model. Additionally, the growth performance of broiler chickens raised in commercial conditions undergoing a natural enteric challenge was evaluated. 

## 2. Materials and Methods

### 2.1. Animal Ethics

Two animal trials were designed and conducted in accordance with animal care committees. The study 1 was performed at the Research Center for Animal Nutrition (DSM Nutritional Products, Village-Neuf, France) according to Directive 2010/63/EU of 22 September 2010 and met the official French guidelines for experiments with live animals. Study 2 was performed at the Ridley Research Center, Australia according to the Prevention of Cruelty to Animals Act (1986), the associated Regulations (2008), and the Victorian ‘Code of Accepted Farming Practice for the Welfare of Poultry’.

### 2.2. Trial 1

#### 2.2.1. Animals and Intestinal Inflammation Model

Day-old male broiler chickens (Ross 308) were supplied by a commercial hatchery (Joseph Grelier S.A., Elevage avicole de la Bohadière, Villemareuil, France). On the day of arrival (day 1), chicks were housed in an environmentally controlled room. The room temperature was adapted to the age of the birds. In the first week, a basal control diet (Table 1) was offered to the birds as crumbled pellets, and afterwards as pelleted feed. Birds had ad libitum access to feed and water. On day 8, animals were moved to an experimental room for randomization. Chickens were allocated by weight into three treatments with 2 cages per treatment, 8 birds per cage, and 16 birds/treatment, which had ad libitum access to feed and water.

The intestinal inflammation model consisted of a challenge diet containing rapeseed meal and potato protein (Table 1) to induce intestinal inflammation due to the presence of antinutritional components [15] and was supplied to treatments B and C from day 8 to 28. On day 14, a 10× dose of a commercial coccidiosis vaccine (Paracox^®^-5, MSD Animal Health, Kenilworth, NJ, USA) was also applied by oral gavage individually to all animals in treatment groups B and C. One vial of 4 mL of vaccine was diluted in 60 mL of water and each bird received 0.6 mL. The vaccine used in the present study is a live and attenuated oral vaccine containing oocysts of *Eimeria acervulina*, *E. brunetti*, *E. maxima*, *E. mitis*, *E. necatrix, E. praecox*, and *E. tenella*.

#### 2.2.2. Experimental Design

A small-scale, well-controlled study was conducted in a randomized block design with three treatments. Each treatment contained two cages with eight birds per cage (48 birds in total). At each sampling day (day 21 and day 28), one cage of eight birds/treatment was used, and each bird was considered an experimental unit. The three treatments were: Unchallenged Control (UC) treatment, receiving a control diet based on corn-soybean meal from day 8 to 28 without the addition of the test material in the diet, and without coccidia vaccination; Challenged Control (CC) treatment, receiving a challenge diet from day 8 to 28 without the addition of the test material, and with a 10× dose of coccidia vaccine applied at day 14; and the PB treatment, receiving a challenge diet from day 8 to 28 with the addition of PB at 1.3 kg/ton of feed, and with a 10× dose of coccidiosis vaccine applied at day 14.

#### 2.2.3. Sample Collection and Analyses

On d 21 and 28 of age, eight randomly pre-selected chickens per treatment (four from each pen) were sacrificed by cervical dislocation after electric narcosis and biological samples were collected (Table 2). Regarding the intestinal sample collected in the present study, ileum was used due to the lesions caused by the live vaccine and due to the potential dysbiosis in this section that could have been impacted by the infection in the upper parts of the intestine.

#### 2.2.4. Immunoglobulin A and Alpha 1-Acid Gycoprotein

Blood was collected on day 21 and 28 in tubes containing EDTA. After centrifugation, the collected plasma was used for ELISA assays. ELISA assays (Abnova GmbH, Heidelberg, Germany) used were double sandwich ELISAs for chicken immunoglobulin A (IgA) and alpha 1-acid glycoprotein (AGP). Absorbance at 450 nm measured the concentration of the specific protein in the test sample.

#### 2.2.5. Isolation and Phenotyping of Intestinal T Cells

Ileal tissue was incubated with 10 mM DTT for 20 min at 37 °C to remove the mucus layer. Pieces of the tissue were mashed in cold RPMI. After 100 µm nylon filtration, Ficoll was added underneath the cell suspension. After centrifugation, the leukocytes were recovered at the interphase between the two layers. The cells were then washed and numerated to adjust their concentration.

The cell suspensions were adjusted to 1 × 10^7^ cells/mL in staining buffer containing BSA. Moreover, 5 × 10^5^ cells were stained with either 0.5 µg (FITC) or 0.25 µg (PE) of antibody. Cells were washed with staining buffer and were analyzed by cytometer (BD, FACSVerse, Becton Drive Franklin Lakes, NJ, USA) using multiple staining antibodies targeted surface cell markers as described in the list below (Table 3).

#### 2.2.6. Gene Expression

Gastrointestinal functionality and inflammation were investigated by gene expression at day 21 and 28. A GIT functionality array (Table 4) was run on the ileum tissue samples. Total RNA was extracted from tissues (stored at −20 °C in RNA later) by lysing tissue with FastPrep^®^ 24 (MP Biomedicals, Illkirch, France), using the phenol-chloroform method (TRIzol reagent; Invitrogen, Cergy Pontoise, France), followed by purification using RNeasy columns by automated method with the Qiacube HT (Qiagen, Courtaboeuf, France). The concentration of RNA was measured by NanoDrop ND-1000 Spectrophotometer (Thermo Fisher Scientific, Illkirch, France) and the purity was estimated by A260/A280 ratio. RNA integrity was assessed by using the Agilent 2100 Bioanalyzer (Agilent Technologies, Basel, Switzerland). The threshold of the RNA Integrity Number (RIN) was set at 7.5 to validate sufficient quality of the RNAs.

The reverse transcription was performed using RT^2^ First Strand Kit (Qiagen, Courtaboeuf, France) with 500 ng of total RNA. The reaction mix was incubated 5 min at 42 °C for genomic DNA elimination, followed by the reverse transcription 15 min at 42 °C. The inactivation of the enzyme was performed by heating 5 min at 95 °C. The resultant cDNAs were amplified with RT^2^ SYBR Green Mastermixes (Qiagen, Courtaboeuf, France) for real-time PCR. The expression of target genes was normalized with housekeeping genes H6PD (Hexose-6-phosphate dehydrogenase) and TBP (TATA box binding protein).

The thermal cycling was run on Light Cycler 96 (Roche Diagnostics, Meylan, France) with the following program: 95 °C 10 min, followed by 40 cycles of denaturation at 95 °C 15 s and hybridization/elongation at 60 °C 1 min. The Delta Ct method was used to determine expression of target genes [16].

#### 2.2.7. Liver and Ileal Histology

Sub-samples of the liver and ileum were collected in 10% formalin. Twenty-four hours after collection, these samples were transferred into 50% ethanol and were processed within a week. Samples were embedded in wax and cut into 5 μm sections. Sections were stained with a hematoxylin and eosin (H&E) staining for ileum and liver and Alcian Blue (mucus staining, pH 2.5) for the ileum. Pictures were taken with an Axio Observer A1 hmicroscope (Carl Zeiss Microscopy, Heidelberg, Germany). Histological pictures were analyzed using AxioVision 4 software [17]. Goblet cells were identified by Alcian Blue staining, imaging, and villi isolation, after which the number of blue pixels per villus was counted. For each villus, the density of goblet cells was calculated by dividing the number of blue pixels by the total number of pixels in the corresponding villus. For both villi length and goblet cells, 8 slides per group were used with at least 10 randomly selected villi per bird for measurement. The liver histology scores are based on non-alcoholic fatty liver disease histopathology scoring [18]. The scoring system was set up within the study, scoring mostly based on tissue damage, hepatocyte ballooning, and immune cell infiltration. The healthiest livers were scored as 1 and the most damaged livers as 5. Liver scoring was conducted blindly and repeated by two independent researchers.

#### 2.2.8. Statistical Analysis

Statistical analyses were performed using Graphpad Prism. Unpaired *t*-tests were performed to determine the differences between the UC and CC chickens. A one-way ANOVA test with a Tukey’s test was used to evaluate the significant differences between group means. Statistical test used are indicated in each results figure and table. In all instances, differences were reported as significant at *p* < 0.05. For all tests, eight replicate birds per treatment were used.

### 2.3. Trial 2

#### 2.3.1. Animals, Diets, and Experimental Design

In total, 360, 1-day-old male broiler chicks (Ross 308) were assigned in a completely randomized design with 3 dietary treatments, 8 replicates per treatment, and 15 birds per replicate. The treatments were either a basal diet (Table 5) without any additives as control, the basal diet supplemented with antibiotic growth promoter (Avilamycin at 10 ppm), or the basal diet supplemented with 0.9 kg/ton of PB. Diets were formulated to meet Ross 308 nutritional recommendations and prepared in three phases: starter (1–10 days), grower (10–24 days), and finisher (25–28 days; Table 5).

The additives were added and mixed homogenously to the basal diets. All diets were steam pelleted and starter diets were further crumbled to maximize feed intake (FI). Feed and water were provided ad libitum. Birds were raised on reused litter from farms previously known to have had enteric challenge. The used litter was topped up with fresh wood shavings to a depth of 3 cm prior to arrival. Chicks were individually weighed on arrival (37 ± 0.5 g), and subsequently, pen body weight (BW) and FI were determined weekly. Feed conversion ratio (FCR) was calculated and corrected for any mortality and common BW. The Return of Investment (ROI) was calculated based on the results obtained from day 1 to 28. The study was finalized at day 28 when the entire intestine from two birds per replicate was scored for lesions characteristic of necrotic enteritis. Lesions were scored based on a 0–4 scale at each segment of the intestine, and then added up for the entire intestine.

An *Eimeria* oocysts count was performed in the excreta of the birds at day 28, and the oocyst count was above 4000 OPG for all treatments.

#### 2.3.2. Statistical Analysis

The data obtained were checked for normality and any outliers were removed prior to statistical analysis. Performance data were then subjected to one-way ANOVA with a Tukey’s test to evaluate the significant differences between group means by using JMP (15.0). Statistical test used are indicated in each results figure and table. In all instances, differences were reported as significant at *p* < 0.05.

## 3. Results

### 3.1. Trial 1

**Figure 1:**
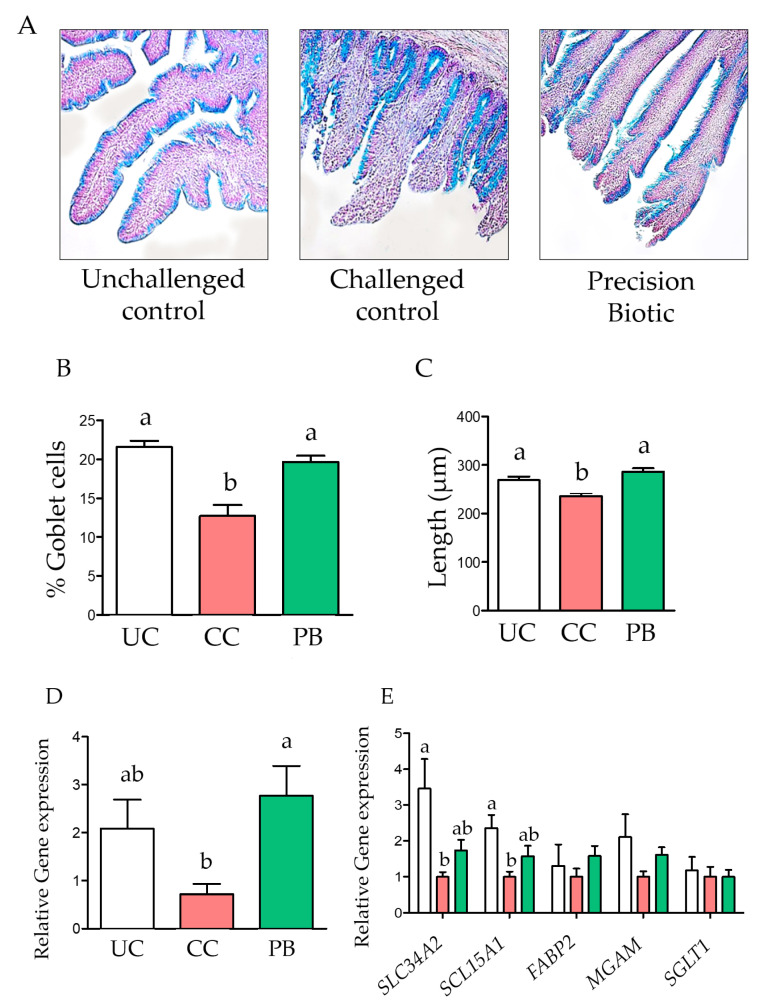
A precision biotic decreased damage in villi caused by an intestinal inflammation model at 21 days of age. (**A**) Representative histology slides of ileal mucosa (20× magnification). (**B**) Percentage of goblet cells, stained with Alcian Blue. (**C**) Mean villi length. (**D**) Relative gene expression of the sodium coupled monocarboxylate transporter 1 (*SLC5A8*) in ileal mucosa. (**E**) Relative gene expression of the phosphorus transporter NaP IIb (*SLC34A2*), the peptide transported PepT1 (*SCL15A1*), the fatty acid binding protein 2 (*FABP2*), maltase (*MAGM*), and the Na+-D-glucose cotransporter (*SGLT1*) in ileal mucosa. One-way ANOVA with Tukey post-hoc test has been performed on all results; statistically significant differences (*p* < 0.05) are indicated by different letters on the bars. UC: unchallenged control; CC: challenged control; PB: precision biotic.

In the current study, the intestinal inflammation model applied (nutritional plus 10× coccidiosis vaccination) induced clear villi damage in the histology analysis, leading to a reduced percentage of goblet cells and the mean villi length in ileal mucosa compared to the UC treatment (Figure 1). However, the dietary supplementation of PB to challenged birds increased both goblet cell counts and villi length compared to the CC treatment, to similar levels as UC, indicating less damage of the villi tips in this group.

Changes in the expression of the sodium coupled monocarboxylate transporter 1 gene (*SLC5A8*; Figure 1) indicate a strong response in the ileal mucosa to the supplementation of PB versus the CC, which was not significantly different from the UC treatment. Among other target genes evaluated in the ileal mucosa, two genes (NaP IIb (*SLC34A2*) and the peptide transporter PepT1 (*SCL15A1*)) were downregulated in the CC treatment compared to the UC (Figure 1), but they were not different from the CC when the birds were supplemented with PB.

**Figure 2:**
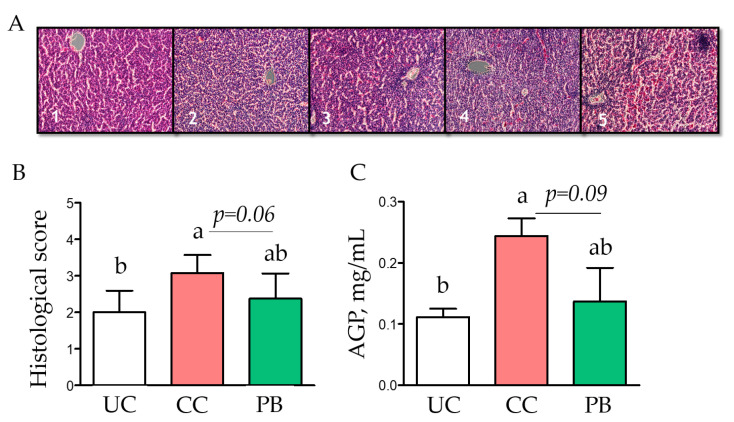
The systemic response to challenge was ameliorated by a dietary precision biotic. (**A**) Representative slides of the liver histology scoring system (20× magnification). (**B**) Mean liver histology scores of 8 chickens per treatment at 28 days of age. (**C**) Concentration of alpha 1-acid glycoprotein (AGP) in the plasma of chickens at day 28 measured by ELISA. One-way ANOVA with Tukey post-hoc test has been performed on all results; statistically significant differences (*p* < 0.05) are indicated by different letters on the bars and trends are indicated by *p*-values. UC: unchallenged control; CC: challenged control; PB: precision biotic.

At day 28 of age, 14 days after the coccidiosis vaccine challenge, liver histology showed increased levels of damage in the CC vs. UC (Figure 2); however, the addition of PB exhibited a trend towards a reduced damage score (*p* = 0.06). Additionally, the CC had a greater concentration of AGP in plasma compared to the UC treatment (Figure 2), but the PB supplementation showed a trend (*p* = 0.09) to reduce its concentration.

**Figure 3:**
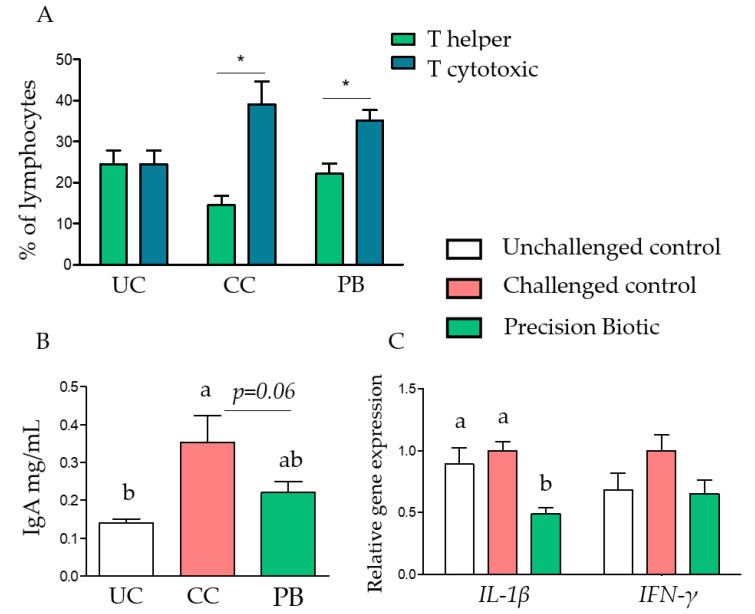
Intestinal inflammation was attenuated by dietary precision biotic at 28 days of age. (**A**) T helper (CD4) and T cell cytotoxic population counts in intestinal tissue samples as a percentage of total lymphocytes in broiler chickens. (**B**) IgA concentrations in plasma measured by ELISA. (**C**) Relative gene expression of *IL-1β* and *IFN-γ* in ileal mucosa of broiler chickens. One-way ANOVA with Tukey post-hoc test has been performed on all results; statistically significant differences (*p* < 0.05) are indicated by different letters or stars on the bars and trends are indicated by *p*-values. UC: unchallenged control; CC: challenged control; PB: precision biotic.

A clear increase in T cytotoxic cells in the intestinal tissue was evident in the challenged groups versus UC treatment at day 28 (Figure 3). Additionally, circulating IgA levels increased in the CC versus the UC treatment, with the PB treatment showing intermediate values and a trend (*p* = 0.06) to reduce the IgA level versus the CC (Figure 3). Furthermore, the expression of *IL-1β*, a pro-inflammatory cytokine, was not significantly affected by the challenge (CC vs. UC), but was significantly reduced by PB supplementation compared to both the UC and the CC. Although not significant (*p* > 0.05), the supplementation of PB also showed a trend towards a downregulation of *IFN-γ* on day 28.

**Figure 4:**
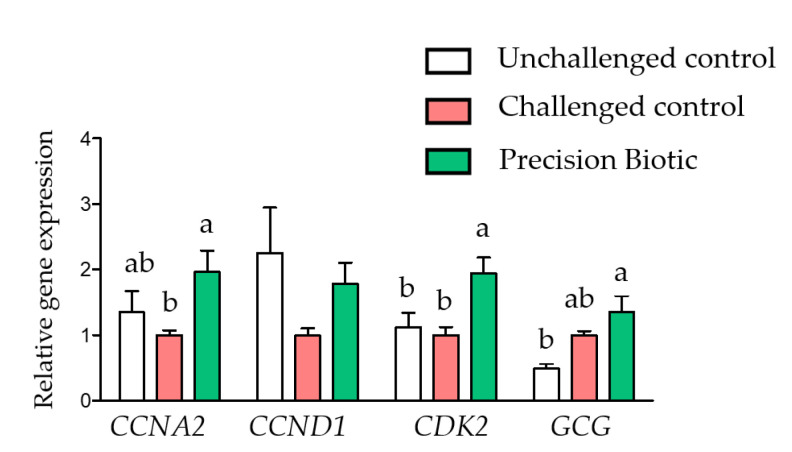
A precision biotic influenced expression of ileal cell cycling gene markers at 21 days of age. Relative expression of genes involved in cell cycling (*CCNA1*, *CCND1*, and *CDK2*) and the pro-glucagon gene GDG that encodes for glucagon and glucagon-like peptides. One-way ANOVA with Tukey post-hoc test has been performed on all results; statistically significant differences (*p* < 0.05) are indicated by different letters on the bars.

Changes in the expression of cell cycling gene markers in the ileal mucosa (Figure 4) in response to PB supplementation were particularly important, with the PB treatment increasing the expression of the *CCNA2* and the *CDK2* genes compared to the CC treatment. Equally important was the increase in the relative expression of the pro-glucagon gene (*GCG*) in response to PB.

### 3.2. Trial 2

The growth performance results of Trial 2 are shown in Table 6. It was observed that the cFCR of the birds at day 28 was significantly affected by the treatments (*p* = 0.04), wherein the birds supplemented with PB showed similar cFCR to birds fed Avilamycin and both were better when compared to control non-supplemented birds. An ROI of 22.5 was observed with the supplementation of PB vs. the UC group.

In Table 7, the lesion scores from each segment of the intestine are shown. It was observed that the supplementation of PB promoted a similar effect to Avilamycin in the duodenum (*p* = 0.007) and ileum (*p* = 0.005) and when considering the whole intestine (*p* = 0.005), while the control non-supplemented group presented a higher score. Additionally, in Table 7 the ileal morphology results are presented, and it was observed that PB and Avilamycin showed similar results for mucosal thickness (*p* = 0.04) and villus length (*p* = 0.03).

## 4. Discussion

The present study assessed the effects of a PB selected for its ability to increase microbial metabolic pathways related to propionate and butyrate biosynthesis, modulation of amino acid degradation and amine metabolism on enterohepatic health markers, and performance of broiler chickens undergoing intestinal challenges. In general, it was observed that broiler chickens supplemented with a PB and undergoing a controlled intestinal inflammation model imposed by nutritional challenge and high dose of coccidiosis vaccination (Trial 1) showed an improved intestinal morphology, improved expression of nutrient transporter genes, lower expression of IL-1β, and lower plasma concentration of AGP. On the other hand, when broilers undergoing a natural challenge (dietary formulation and used litter from poultry farms known for having enteric disease outbreak), the supplementation of PB significantly improved the growth performance as well the intestinal health similarly to Avilamycin.

The ability of potato protein to induce intestinal dysbacteriosis, ileal mucosa and liver lesions, and performance losses in broilers have been reported [15]. Similarly, dietary challenges in combination with coccidiosis vaccines at high doses have been used as a model of enteric dysbiosis and gut barrier failure in broilers [19], which aim to simulate challenges that broiler chickens encounter in commercial production systems in the presence of predisposing factors such as coccidiosis, poor quality feed ingredients, and mycotoxin contaminations in feed. The presence of a nutritional challenge and high coccidiosis vaccine inoculation induced clear intestinal damage. Significant reductions in the counts of goblet cells in intestinal villi in the presence of coccidiosis challenge have been reported [20]. In the current study, the ileal villi exhibited a reduced number of goblet cells surrounding the tip of the villi, which coincides with the changes observed by Collier et al. [21] in broiler chickens challenged with *Eimeria maxima* and *Clostridium perfringens*.

Dietary supplementation of PB to challenged birds increased both goblet cell counts, and villi length compared to the challenge control treatment. Even though the present study did not evaluate changes in the mucosal or luminal bacterial communities, the selection of PB as a microbiome metabolic modulator targeting short chain fatty acid (SCFA) and nitrogen microbial pathways would suggest that this effect was driven by shifts in microbiome metabolism and metabolites that could affect the expression of immune-related genes to enhance intestinal morphology; however, this effect needs to be evaluated in further in vivo studies. Furthermore, changes in the expression of *SLC5A8* indicate a strong response in the mucosa to the supplementation of PB. *SLC5A8* is an Na+ coupled co-transporter and one of the two reported transporters of SCFA in the intestinal mucosa [22]. Via this transporter, SCFA transport also stimulates the absorption of Na and water, which has been suggested as one of the compensatory mechanisms by which production of SCFA in the intestine preserves the homeostasis in the presence of an enteric insult [23]. More importantly, this protein can increase the passage of SCFA from the intestinal lumen to the cytosol of the intestinal epithelial cells, where butyrate is used as a source of ATP [23], supporting cellular homeostasis during enteric stress [24].

Additionally, the two genes that were downregulated in the challenged group were the phosphorus transporter NaP IIb (*SLC34A2*) and the peptide transporter PepT1 (*SCL15A1*). An increase in the expression of PepT1 in the duodenum and ileal mucosa has been observed in chickens infected with *Eimeria acervulina* or *E. maxima* [25], whereas Adedokun et al. [26] reported an increased expression of NaPIIb in the duodenum of chickens challenged with a coccidiosis vaccine. Villus damage can affect the expression of transporters located in the brush border, and in some cases, it may elicit compensatory mechanisms of absorption. Other brush border genes measured in the current study, such as fatty acid binding protein 2 (*FABP 2*), maltase (*MGAM*), and the Na+-D-glucose cotransporter *SGLT1*, did not show significant differences among the three groups. Samples in this study were taken in the ileum, contributing to a lower sensitivity of nutrient absorption genes compared to the duodenum and jejunum. For instance, Tan et al. [20] reported a reduction in maltase activity in the jejunum of chickens in response to a coccidia vaccine challenge.

Liver histology showed increased damage in response to the challenge as measured by a scoring system [18], and the supplementation of PB partially reduced this damage. Previously, Palliyeguru et al. [15] reported that a dietary challenge with potato protein in chickens induced a greater prevalence of liver lesions compared to soy-based diets. Furthermore, the challenged increased plasma concentration of AGP, and the supplementation of PB tended to reduce this effect, further corroborating its effect on liver health. AGP is one of the major acute phase proteins and its serum concentration increases in response to systemic tissue injury, inflammation, or infection [27]. In chickens, increments in the concentration of AGP have been associated with increased blood mononuclear cell proliferation [28]. Increased AGP concentration in the blood of chickens have been reported with direct injections of *Escherichia coli* LPS [28] as well as coccidiosis related challenges [29]. The disruption of the intestinal mucosa by coccidiosis is likely to have facilitated a systemic AGP response in the current study, which was partially ameliorated by the PB supplementation.

A clear increase in T cytotoxic cells was observed in the intestinal tissue in response to the challenge. Increased numbers of cytotoxic T cells expressing the CD8 cell surface antigen have been reported after primary infections with *Eimeria*, which is also linked to increased production of *IFN-γ* [30]. The supplementation of PB did not reduce cytotoxic T cells but showed a trend to reduce the expression of *IFN-γ* in ileum on day 28. On the other hand, PB significantly downregulated the expression of *IL-1β* in the ileum on day 28. Avian *IL-1β* cytokine acts as a rapidly induced pro-inflammatory mediator in response to bacterial, viral, and parasite challenges [31]. The modulatory response of PB may reflect changes in the microbial pathways, reducing the dysbacteriosis pressure in both challenged and unchallenged birds.

Beneficial effects on the expression of cell cycling gene markers in the ileal mucosa in response to PB were also observed. Precision biotic supplementation increased the expression of the *CCNA2* and the *CDK2* genes. Profound changes in gene expression of cyclins and cyclins-dependent kinases have been reported in response to increased butyrate concentrations in cell culture models [32,33]. Similarly, Yin et al. [34] found changes in the expression of these genes in the ileum of young chicks exposed to bacterial inoculum that exhibited different colonization rates. Those reported findings suggest that this set of genes is important in the interaction between bacterial colonization and mucosal development in chickens. The increase in cell cycle might contribute to the improvements seen in villi histology, leading to more resilience to enteric stress. The mechanism behind the effects of PB on cell cycling genes and its relevance in intestinal health needs further evaluation. Nevertheless, the increase in the relative expression of *GCG* by PB is another aspect that deserves further discussion. This gene encodes for glucagon and glucagon-like peptides *GLP-1* and *GLP-2* in chickens [35], which play an important role in energy balance through regulation of glucose, lipid, and amino acid metabolism (glucagon), control of gastric emptying and food intake (*GLP-1*), and potentially maintaining epithelial cell integrity (*GLP-2*) [36]. Since the present study did not measure the concentration of these proteins, inferences on post-translational modifications cannot be drawn. However, these findings suggest the potential of PB microbiome metabolic modulators to regulate local and systemic metabolism in birds.

The results obtained in Trial 1 may further explain the results found in Trial 2, wherein the supplementation of PB mitigated the negative impact of an enteric challenge. In trial 2, broiler chickens were fed a diet formulated to provide a nutritional challenge (Canola, barley, and meat meals) and raised under used litter from poultry farms known for having coccidiosis outbreak. It was clearly demonstrated the beneficial effect of PB on the growth performance of the birds, which was statistically comparable to Avilamycin, and on the intestinal health as measured by intestinal lesion score and ileal morphology. These results agree with those of Walsh et al. [4] and Jacquier et al. [5], who reported the consistent effect of this PB in improving the growth performance of broiler chickens mainly through the modulation of microbial metabolic pathways.

## 5. Conclusions

In conclusion, the novel PB tested herein demonstrated to have a positive effect on the integrity of the intestinal mucosa of broiler chickens undergoing a challenge to stimulate intestinal inflammation. Additionally, it showed a positive effect on the growth performance and intestinal health of chickens raised under commercial conditions and fed a commercial type of diet. Changes in the gene expression of an SCFA transporter and cell cycling genes suggest that structural effects in the intestine may be mediated by microbial changes in the production of SCFA and nitrogen metabolites, and changes in the expression of the pro-glucagon gene in the ileum suggest the potential for local and systemic effects on the host metabolism, likely through the production of microbial metabolites that need to be further evaluated. A downregulation of *IL-1β* in the ileal mucosa and a trend for a reduction in the concentration of liver AGP indicated a greater ability of supplemented birds to modulate their immune function, locally and systemically, in the presence of an inflammatory insult in the intestine. 

## Figures and Tables

**Table 1:** Ingredient composition and nutritional specifications of experimental diets (Trial 1).

Ingredient, %	Basal Diet	Challenge Diet
Corn	55.40	65.80
Soybean meal	38.75	-
Rapeseed meal	-	13.00
Potato protein	-	16.00
Soya oil	2.00	1.50
Calcium carbonate	0.40	0.15
Dicalcium phosphate	2.00	2.00
NaCl	0.20	0.20
DL-Methionine	0.20	0.10
L-Lysine	0.09	0.20
Vitamin-mineral premix ^1^	1.00	1.00
Coccidiostat ^2^	0.06	-
Calculated Nutrient Composition		
AME (kcal/kg)	2990	3083
Crude protein (%)	22.0	21.9
Lysine (%)	1.27	1.25
Cysteine + Methionine (%)	0.87	0.90
Threonine (%)	0.84	1.07
Total P (%)	0.74	0.73
Total Ca (%)	0.96	0.92
Analyzed Protein Composition		
Crude protein (%)	23.3	20.9

^1^ The premix provided per kilogram of diet: vitamin A: 10,000.0 IU; vitamin E: 40.0 mg; vitamin K3: 3.0 mg; vitamin C: 100.0 mg; vitamin B1: 2.5 mg; vitamin B2: 8.00 mg; vitamin B6: 5.0 mg; vitamin B12: 0.03 mg; niacin: 50.0 mg; pantothenate calcium: 12.0 mg; folic acid: 1.50 mg; biotin 0.15 mg; cholin: 450.0 mg; ethoxyquine: 54.0 mg; sodium: 1.17 g; magnesium: 0.8 g; manganese: 80 mg; iron: 60 mg; copper: 30 mg; zinc: 54 mg; iodine: 1.24 mg; cobalt: 0.6 mg; selenium: 0.3 mg. ^2^ Avatec ^®^, Zoetis, Parsippany, NJ, USA.

**Table 2:** Biological samples, bird age of collection, and analyses performed.

Sample	Age	Analyses
Blood (plasma, serum)	day 21 and 28	ELISA: IgA, and AGP
Ileum tissue	day 21 and 28	Gene expression Morphology, crypt villus length
Liver	day 21 and 28	Morphology Pathology
Ileum Tissue	day 21 and 28	T Cell phenotyping

**Table 3:** Target phenotypes used in analysis of intestinal T cells.

Cell-Surface Marker	Target	Reference
CD45	Leukocytes	MCA2413PE Biorad
CD3	T cells	MA5-28694 Invitrogen
CD4	T-helper cells	MCA2164F Biorad
CD8a	Cytotoxic T cells	MA5-28725 Invitrogen
CD8b	Cytotoxic T cells	C2259-99N US Biologica

**Table 4:** Target genes, functions, and primer sequences used for qPCR in ileum samples.

Gene	Function	Primer Sequences (5′→3′)
*IL-1β*	Pro-inflammatory	Primer sequences provided by Qiagen, Courtaboeuf, France
*IFN-γ*	Pro-inflammatory	
*SLC5A10*	Glucose transporter (SGLT1)	
*SCL15A1*	Peptide transporter	
*SLC34A2*	Phosphorus transporter	
*SLC5A8*	Sodium coupled monocarboxylate transporter 1	F: GGT-GGG-ACC-TTC-ACA-TGG-AC R: AGA-GGG-ACA-TTT-TTG-CGT-GG
*SGLT1*	Na+-D-glucose cotransporter	F: TGG-TTG-TTC-TAG-GAT-GGG-TG R: CAG-TGA-CAG-CAT-CTC-GGA-AG
*CCNA2*	Cyclin A1	F: TTG-CCT-CAT-GGA-CCT-TCA-CA R: GCA-TGG-TAC-TTT-GTG-CTC-TTG-T
*CCND1*	Cyclin D1	F: CAC-TTG-GAT-GCT-GGA-GGT-CTG R: CGA-ACG-ACA-AAA-ACC-TGT-CCA
*CDK2*	Cyclin dependent kinase 2	F: ATT-TTT-GCT-GAG-ATG-GTG-ACG-C R: ACG-TGC-GGA-AGA-TAC-GGA-AG
*GCG*	Pro-glucagon	F: TCC-AGA-ACA-TGG-GAA-CAG-AGA R: CTG-TAT-GCC-AGA-CTT-CCA-TTG-T

**Table 5:** Ingredient composition and nutritional specifications of experimental diets (Trial 2).

Ingredient, %	Starter 1–10 Days	Grower 10–24 Days	Finisher 24–48 Days
Wheat ^1^	55.88	55.63	58.22
Soybean meal	28.12	22.56	17.24
Canola meal	4.25	6.00	7.00
Meat meal	4.00	3.20	2.52
canola oil	2.60	3.55	4.08
Barley	2.00	4.00	5.00
Canola seed	1.00	3.00	4.00
Limestone fine	0.733	0.753	0.770
Salt	0.316	0.283	0.203
DL-Methionine	0.293	0.247	0.213
HCL-Lysine	0.261	0.239	0.236
Vitamin-Mineral Premix ^2^	0.200	0.200	0.200
L-Thr	0.143	0.111	0.088
Na Bicarbonate	0.103	0.120	0.135
Choline Chloride (70%)	0.050	0.050	0.050
Protease	0.020	0.020	0.020
Phytase	0.020	0.020	0.020
Carbohydrase	0.010	0.010	0.010
Calculated nutrient composition			
AME (Kcal/kg)	2990	3100	3180
Crude Protein (%)	23.1	21.3	19.4
Dry Matter (%)	90.80	90.84	90.83
Dig Lys (%)	1.274	1.153	1.027
Dig Met (%)	0.603	0.543	0.492
Dig M + C (%)	0.945	0.873	0.805
Dig Thr (%)	0.856	0.773	0.685
Calcium (%)	0.900	0.850	0.800
Av P (%)	0.450	0.425	0.400
Na (%)	0.220	0.210	0.180
Cl (%)	0.330	0.302	0.249
K (%)	0.833	0.775	0.711

^1^ Tested substance were added in substitution of wheat. ^2^ The premix provided per kilogram of diet: vitamin A: 10,000.0 IU; vitamin E: 40.0 mg; vitamin K3: 3.0 mg; vitamin C: 100.0 mg; vitamin B1: 2.5 mg; vitamin B2: 8.00 mg; vitamin B6: 5.0 mg; vitamin B12: 0.03 mg; niacin: 50.0 mg; pantothenate calcium: 12.0 mg; folic acid: 1.50 mg; biotin 0.15 mg; cholin: 450.0 mg; ethoxyquine: 54.0 mg; sodium: 1.17 g; magnesium: 0.8 g; manganese: 80 mg; iron: 60 mg; copper: 30 mg; zinc: 54 mg; iodine: 1.24 mg; cobalt: 0.6 mg; selenium: 0.3 mg.

**Table 6:** Growth performance of broiler chickens from 1 to 28 days of age according to the experimental treatments (Trial 2).

Treatment	BWG, g	cFCR	ROI
Control	1372	2.236 ^a^	-
Avilamycin	1451	1.917 ^b^	-
Precision biotic	1431	1.929 ^b^	22.5
SEM	28.8	0.11	
*p* Value	0.15	0.04	

^a,b^ values in a row column with no common superscripts differ significantly at *p* < 0.05. Mean values are based on 8 replicates/treatment of 15 birds per replicate. BWG: body weight gain; cFCR: corrected feed conversion ratio (corrected for common body weight of the strain at the specific age); ROI: Return of Investment (ratio between net profit by its cost).

**Table 7:** Intestinal lesion score and ileal morphology of broiler chickens at 28 days of age according to the experimental treatments (Trial 2).

	Lesion Scores ^1^			
Treatment	Duodenum	Ileum	Caeca	Whole Intestine
Control	3.56 ^a^	1.50 ^a^	0.25	5.31 ^a^
Avilamycin	1.00 ^b^	0.31 ^b^	0.63	1.94 ^b^
Precision biotic	0.81 ^b^	0.62 ^ab^	0.69	2.13 ^b^
SEM	0.42	0.32	0.32	0.66
*p* Value	0.007	0.005	0.16	0.005
	Ileal Morphology			
Treatment	Mucosa Thickness (µm)	Villus Length (µm)	Crypt Depth (µm)	Villus/Crypt
Control	584.7 ^b^	323.5 ^b^	246.5	1.380 ^b^
Avilamycin	774.4 ^a^	517.7 ^a^	242.2	2.349 ^a^
Precision biotic	782.4 ^a^	461.1 ^a^	306.4	1.633 ^b^
SEM	48.4	45.8	17.6	0.22
*p* Value	0.02	0.04	0.06	0.03

^1^ Lesions were scored based on a 0–4 scale at each segment of the intestine, and then added up for the entire intestine. ^a,b^ values in a row column with no common superscripts differ significantly at *p* < 0.05. Mean values are based on two birds per replicate and eight replicates per treatment.

## Data Availability

The data presented in this study are available on request from the corresponding author.
